# Unexpectedly Low HbA1c in a Patient With Newly Diagnosed Diabetes Mellitus and Thalassemia Trait: A Case Report

**DOI:** 10.7759/cureus.87419

**Published:** 2025-07-07

**Authors:** Samer Alhasan, Azeez Alzafiri, Hisham Tharwat, Ramia Alhasan, Nadia Alrashidi

**Affiliations:** 1 Internal Medicine, Al Jahra Hospital, Al Jahra, KWT

**Keywords:** continuous glucose monitoring, diabetes mellitus, fructosamine, hba1c, hemoglobinopathy, thalassemia trait

## Abstract

Hemoglobin A1c (HbA1c) is widely used to assess long-term glycemic control in patients with diabetes mellitus; however, its reliability may be compromised in certain clinical scenarios, including hemoglobinopathies. We report the case of a 46-year-old man who presented with symptoms of hyperglycemia and was found to have diabetic ketosis. Despite significant hyperglycemia, his HbA1c was markedly low. Further evaluation confirmed the presence of thalassemia trait, which explained the discrepancy due to its impact on red blood cell turnover and HbA1c assay interference. This case highlights the importance of recognizing conditions that can distort HbA1c values and the need to use alternative glycemic monitoring strategies such as continuous glucose monitoring, corrected HbA1c, and serum fructosamine in such patients. Accurate interpretation of glycemic markers in the context of hemoglobinopathies is essential for appropriate diagnosis and effective diabetes management.

## Introduction

Glycated hemoglobin (HbA1c) reflects average blood glucose levels over approximately two to three months and is widely regarded as the gold standard for monitoring long-term glycemic control in patients with diabetes mellitus [1]. Its utility lies in its ability to predict the risk of chronic diabetic complications and reduce the need for frequent glucose testing. However, the accuracy of HbA1c can be compromised by several factors unrelated to glycemia, particularly conditions affecting red blood cell (RBC) lifespan and structure [2,3]. Hemoglobinopathies, including thalassemia traits, are among the most significant confounders of HbA1c interpretation. In these conditions, altered erythrocyte turnover and the presence of abnormal hemoglobin variants can lead to either falsely low or falsely elevated HbA1c values, depending on the assay method used and the type of hemoglobin abnormality present [4-6]. This can result in the underdiagnosis or delayed treatment of diabetes, especially in patients who appear clinically hyperglycemic despite a “normal” or low HbA1c. Thalassemia traits are particularly common in regions with a history of endemic malaria. In Kuwait, the prevalence of beta-thalassemia trait is estimated at 2.12%, while alpha-thalassemia trait may affect up to 40% of the population [7,8]. These figures are substantially higher than global averages, where the beta-thalassemia carrier rate is approximately 1.5% [9]. Given the high local prevalence, physicians practicing in such regions must remain vigilant to the potential for misleading HbA1c results. We present a case of new-onset diabetes mellitus in a middle-aged Kuwaiti man who presented with classic hyperglycemic symptoms and biochemical ketosis, yet had a markedly low HbA1c level. Subsequent workup revealed thalassemia trait, underscoring the limitations of HbA1c in such contexts and highlighting the need for alternative monitoring strategies such as continuous glucose monitoring (CGM) and serum fructosamine.

## Case presentation

A 46-year-old Kuwaiti male with no known past medical history presented to the emergency department of New Jahra Hospital with a two-month history of polyuria, polydipsia, unintentional weight loss (~5 kg), fatigue, dry mouth, and nocturia. Two days prior to presentation, he developed low-grade fever, sore throat, and a non-productive cough. He denied any recent travel, medication use, or family history of diabetes or hemoglobinopathies. On initial assessment, the patient appeared euvolemic but clinically hyperglycemic. His capillary blood glucose level was markedly elevated (25 mmol/L). Urinalysis revealed 3+ glucose and positive ketones. Arterial blood gas showed mild compensated metabolic acidosis. There were no signs of infection or dehydration requiring urgent hospitalization. He was diagnosed with new-onset diabetes mellitus complicated by mild diabetic ketosis and an upper respiratory tract infection. The patient declined hospital admission but agreed to outpatient management. He was stabilized with intravenous fluids and subcutaneous insulin and was scheduled for close endocrinology follow-up. During follow-up, laboratory results were surprising: his HbA1c was <2%, a value discordant with his hyperglycemic symptoms and ketotic presentation (Table 1). Repeat testing confirmed the result at 2.3%. Further laboratory evaluation revealed microcytic anemia with reticulocytosis and normal bilirubin levels. Hemoglobin electrophoresis confirmed beta-thalassemia trait. Due to the unreliability of HbA1c in this context, CGM was initiated to guide therapy. CGM data correlated with elevated glucose readings and provided a more accurate reflection of glycemic burden. Over the next eight weeks, the patient’s glycemic control improved with basal-bolus insulin therapy.

**Table 1:** Laboratory investigations at initial and follow-up visits HbA1c, glycated hemoglobin

Parameter	Obtained Value	Reference Range
Fasting blood glucose	25 mmol/L	3.9–5.5 mmol/L
Serum ketones	3.0 mmol/L	<0.6 mmol/L
Venous blood gas pH	7.40	7.35–7.45
Bicarbonate	20 mmol/L	22–28 mmol/L
Red blood cells	6.86	4.5–5.5
Hemoglobin	121 g/L	135–175 g/L
Hematocrit	0.372	0.4–0.5
Mean corpuscular volume	54 fL	80–96 fL
Reticulocyte count	2.9%	0.5–2.5%
Total bilirubin	12 µmol/L	5–21 µmol/L
HbA1c (initial)	<2%	4.0–6.0% (non-diabetic)
HbA1c (repeat)	2.3%	4.0–6.0% (non-diabetic)
Hemoglobin electrophoresis	Consistent with beta-thalassemia trait	N/A

**Figure 1:**
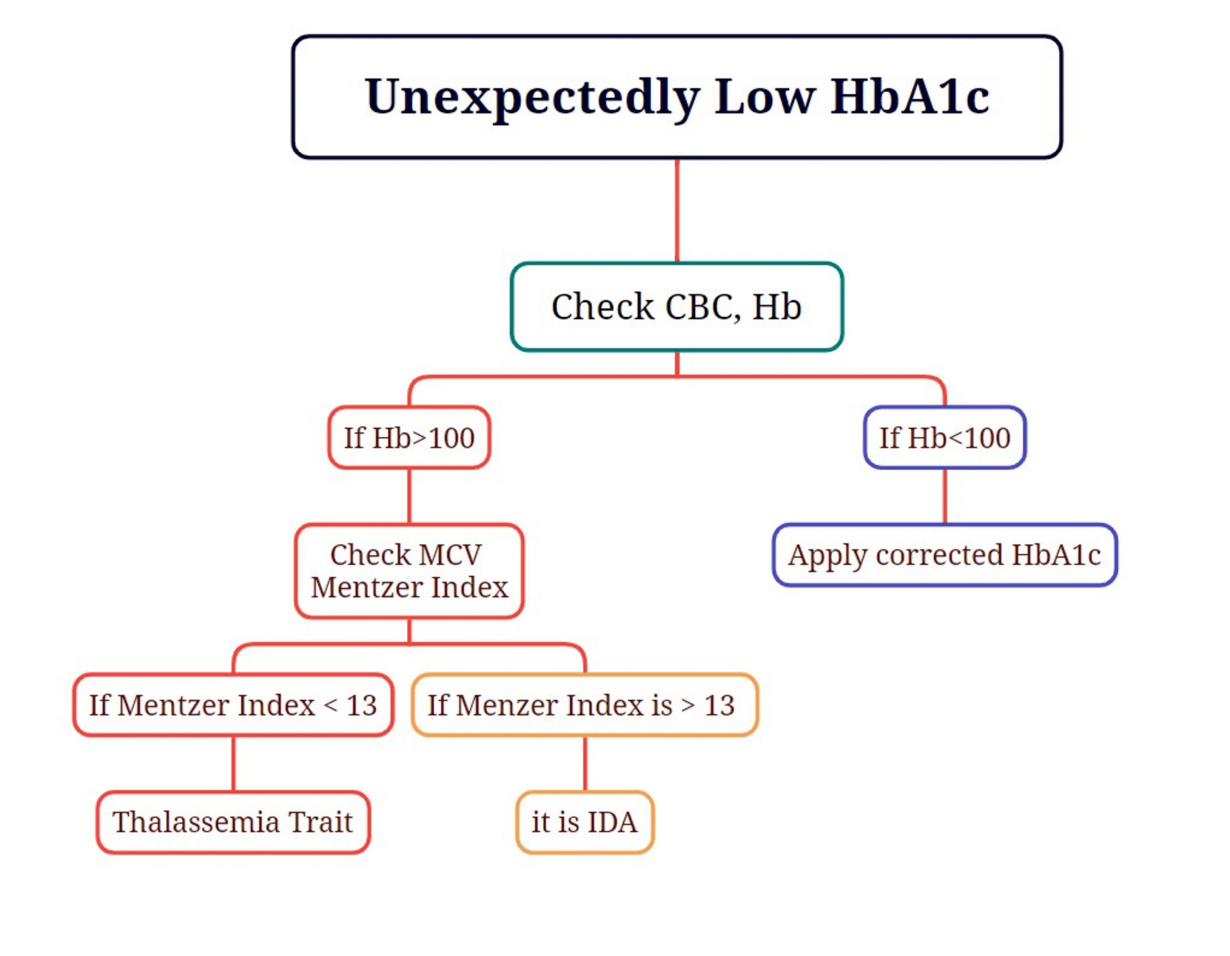
Approach to low HbA1c CBC, complete blood count; Hb, hemoglobin; HbA1c, glycated hemoglobin; IDA, iron deficiency anemia; MCV, mean corpuscular volume

In patients with significant anemia, HbA1c values may be falsely low due to reduced RBC lifespan. Although there is no universally standardized correction method, some clinical studies have proposed regression-based or empirical formulas to estimate a corrected HbA1c. One such formula suggests: Corrected HbA1c = Measured HbA1c + (15 − Hemoglobin in g/dL) × 0.4. This equation is primarily used in research and is not validated for routine clinical application. In clinical practice, CGM or serum fructosamine is preferred for glycemic assessment in patients with anemia or other conditions affecting RBC turnover. In our patient, the hemoglobin concentration was 121 g/L, which falls within the near-normal range and indicates only mild anemia. Therefore, applying a correction formula was not deemed necessary. However, this situation underscores the importance of considering such adjustments in patients with more pronounced anemia and discordant glycemic indicators. Additionally, the presence of microcytosis prompted further evaluation to distinguish between iron deficiency anemia and thalassemia trait. We calculated the Mentzer index, a simple and widely used screening tool in cases of microcytic anemia. It is calculated by dividing the mean corpuscular volume (MCV) by the RBC count. In this patient, the Mentzer index was 54 fL / 6.86 × 10⁶/μL = 7.87. A value below 13 supports the diagnosis of thalassemia trait, whereas a value above 13 is more indicative of iron deficiency anemia. This finding, consistent with hemoglobin electrophoresis results, further supported the diagnosis of beta-thalassemia trait. The Mentzer index is particularly helpful in regions such as Kuwait, where thalassemia is relatively prevalent, and serves as a useful, cost-effective initial diagnostic indicator in patients presenting with unexplained microcytic anemia (Figure 1).

## Discussion

HbA1c remains the primary marker for assessing long-term glycemic control in patients with diabetes mellitus. It reflects the average plasma glucose concentration over the preceding 8-12 weeks, based on the degree of hemoglobin glycation. However, in certain clinical conditions, its reliability is compromised. These include hemoglobinopathies such as thalassemia and sickle cell disease, anemia or hemolysis, recent transfusions, micronutrient deficiencies, and chronic liver or kidney disease. Furthermore, some ethnic populations may exhibit discordant HbA1c-to-glucose relationships, and assay interference, particularly with high-performance liquid chromatography (HPLC), can result in inaccurate values [1-3]. In our case, the patient presented with overt hyperglycemic symptoms and biochemical evidence of ketosis. Surprisingly, his HbA1c was markedly low at 2.3%, a value not reflective of his clinical presentation. This discordance prompted further investigation, ultimately revealing a diagnosis of beta-thalassemia trait. Thalassemia leads to altered erythrocyte turnover and the presence of hemoglobin variants, both of which contribute to falsely low HbA1c readings, especially when measured via ion-exchange HPLC methods [4,5]. Such discrepancies can delay diabetes diagnosis or misguide therapeutic decisions, underscoring the need for critical interpretation of glycemic indices in patients with hemoglobinopathies. A systematic approach is essential when HbA1c does not align with clinical findings. In patients with significant anemia (e.g., hemoglobin <100 g/L), some researchers propose correction formulas such as: Corrected HbA1c = Measured HbA1c + (15 - Hb in g/dL) × 0.4. Although not yet validated for routine clinical use, such estimates may aid interpretation. In our patient, who had only mild anemia (Hb = 121 g/L), correction was not necessary. Nonetheless, clinicians should consider alternative markers when encountering unexpectedly low HbA1c in symptomatic patients. In cases of microcytic anemia, the Mentzer Index - calculated by dividing MCV by RBC count - can help distinguish thalassemia trait from iron deficiency anemia. In this case, a Mentzer index of 7.87 strongly suggested thalassemia trait and guided further diagnostic evaluation. This index remains a practical and cost-effective screening tool, particularly in regions with high thalassemia prevalence such as Kuwait [6-8]. To circumvent the limitations of HbA1c, CGM has emerged as a reliable tool. CGM uses subcutaneous sensors to measure interstitial glucose levels continuously, providing detailed glycemic profiles that include time in range, glycemic variability, and trends over days to weeks. It offers real-time feedback, detects asymptomatic hypoglycemia, and is unaffected by RBC lifespan or hemoglobin variants. In contrast to HbA1c, which provides only retrospective averages, CGM enables individualized and dynamic glycemic management [9]. Fructosamine is another valuable marker in such scenarios. It measures glycated serum proteins, primarily albumin, and reflects average glucose levels over a shorter period (two to three weeks). While less commonly used, it remains a practical alternative when HbA1c is unreliable or when short-term glycemic monitoring is needed, such as in pregnancy or after medication changes. However, its interpretation can be affected by hypoalbuminemia and liver disease [5]. In this patient, CGM was selected due to its real-time data capture and patient engagement advantages. Serial downloads revealed elevated glucose levels that correlated with clinical symptoms, facilitating effective insulin titration and glycemic control. CGM proved to be a valuable monitoring tool in this case, compensating for the unreliability of HbA1c and confirming the patient’s glycemic burden. This case highlights several important clinical implications. First, clinicians should maintain a high index of suspicion for hemoglobinopathies when HbA1c values are discordant with clinical or biochemical evidence. Second, routine use of alternative markers such as CGM and fructosamine should be considered, especially in regions with high prevalence of thalassemia. Third, laboratory interpretation must be integrated with clinical judgment, and reliance on HbA1c alone may lead to underdiagnosis or mismanagement of diabetes. Given Kuwait’s elevated rates of alpha- and beta-thalassemia traits, the risk of misinterpreting HbA1c is significant. Broader access to CGM and increased awareness of its value in such contexts may enhance diagnostic accuracy and therapeutic outcomes. Further research into hemoglobin-independent glycemic markers is warranted to improve care in this vulnerable patient population.

## Conclusions

This case highlights the limitations of relying solely on HbA1c for diagnosing and monitoring diabetes in patients with underlying hemoglobinopathies. The discordance between the patient's significant hyperglycemia and unexpectedly low HbA1c prompted further evaluation, which revealed beta-thalassemia trait. This finding underscores the importance of integrating clinical judgment with appropriate biochemical and hematological investigations, particularly in regions with a high prevalence of thalassemia. Alternative glycemic markers such as CGM or serum fructosamine should be considered when HbA1c values appear inconsistent with the clinical picture. Recognizing these diagnostic pitfalls is essential to avoid misdiagnosis or under-treatment and ensure optimal management of diabetes. The patient’s glycemic status was successfully monitored using CGM, which provided a reliable assessment of his glucose trends. Insulin therapy was adjusted based on CGM data, resulting in improved glycemic control during follow-up visits.
